# Supplementary material for: Debiased ambient vibrations optical coherence elastography to profile cell, organoid and tissue mechanical properties
Source: Commun Biol. 2023 May 18;6:543. doi: 10.1038/s42003-023-04788-0 (PMC10195840; doi:10.1038/s42003-023-04788-0)
Supplement: Supplementary file 1 — Supplementary Information [file 42003_2023_4788_MOESM1_ESM.pdf]

Debiased ambient vibrations optical coherence elastography to profile cell, organoid and tissue mechanical properties

Supplementary files

Comparison between passive elastography OCE and debiaised ambient vibration OCE.

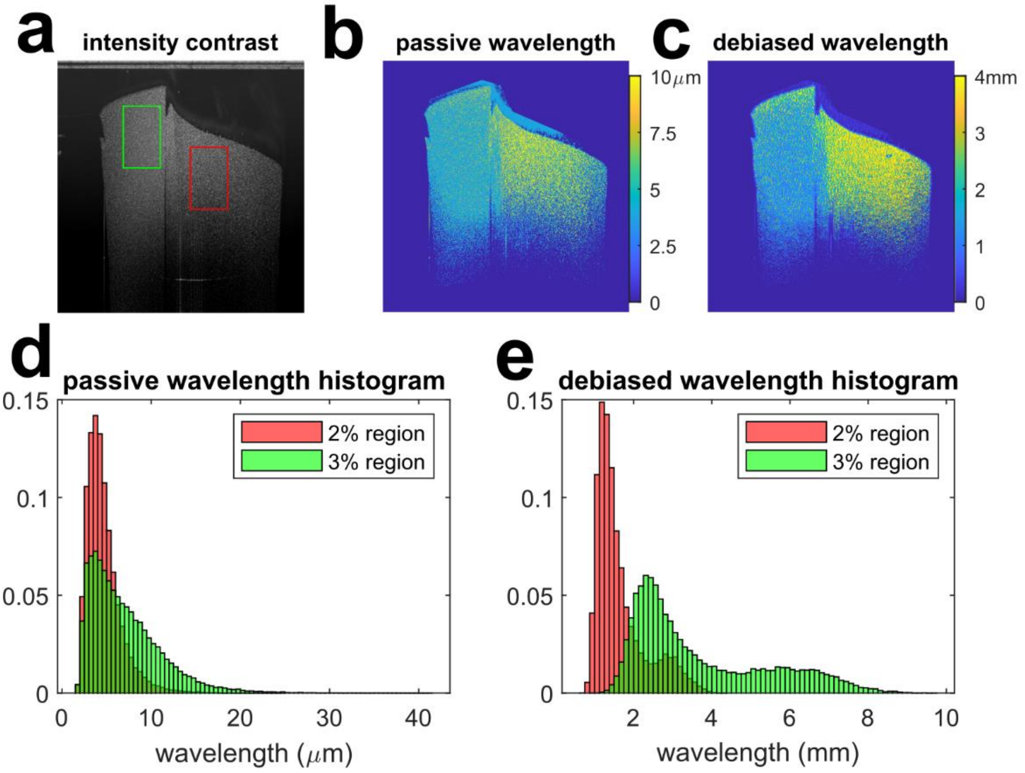

Figure S1: a) Intensity contrast for a hybrid 2/3 % phantom agarose gel with green and squares respectively delimiting regions of interests in the 2% and 3% gels. **b)** Corresponding mechanical contrast and wavelength mapping derived from passive elastography, and **c)** debiased ambient vibration algorithms. Colorbar shows that passive elastography wavelength is order of magnitude lower than from the debiased algorithm which values correlate well with elasticity as measured by state of the art measurements (Fig2c). **d)** Passive wavelength distribution show a large overlap between calculated values in the ROI and an underestimated values as shown in simulation (fig1c,d). **e)** Debiased elastography achieves a better separation between the soft (red) and stiff region (green).

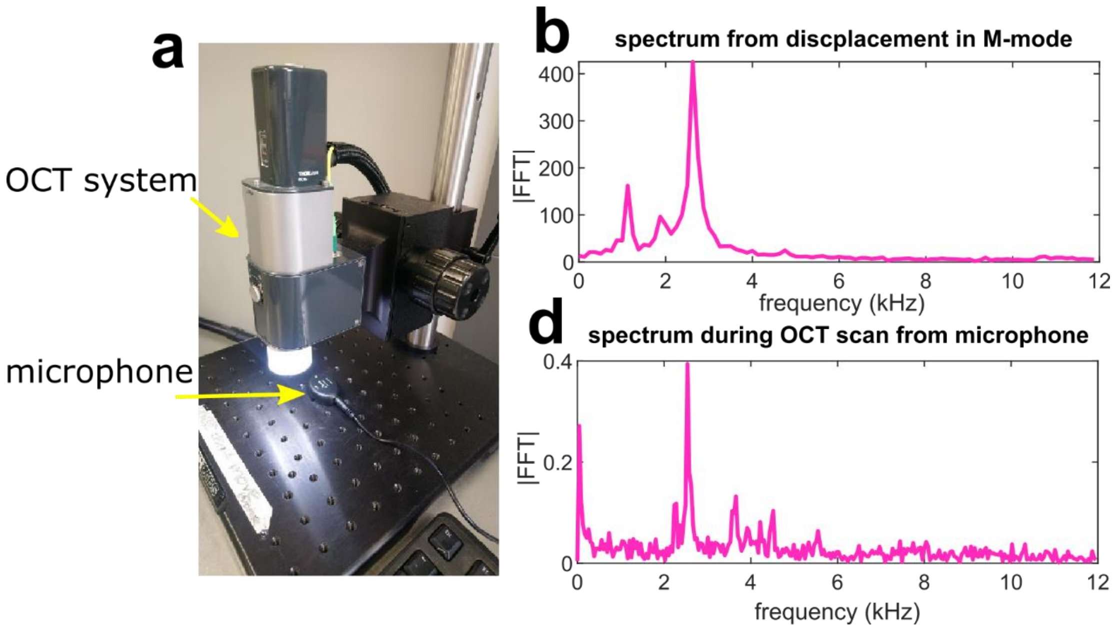

Figure S2: a) Set up for the measurement of local displacement inside an agarose gel sample over time while scanning in M-Mode (i.e. repeat of depth A-scan at same location) to achieve a 48kHz rate, and verified with a contact sensor . **b)** Corresponding OCT M Mode spectra displaying a characteristic peak frequency of 2.5kHz that was characteristic of all samples in our study. **c)** This characteristic frequency was further confirmed by measurements from a contact microphone placed in contact with the OCT base plate and which showed the same characteristic frequency.
